# Comparative genomic insights into a plant-associated *Enterobacter adelaidei* strain isolated from the taro rhizosphere

**DOI:** 10.1186/s12864-026-13081-3

**Published:** 2026-06-17

**Authors:** Sanjoy Kumar Mukharjee, Md. Faruk Hasan, Biswanath Sikdar

**Affiliations:** 1 Department of Microbiology, University of Rajshahi, Rajshahi, 6205 Bangladesh; 2 Department of Microbiology, Noakhali Science and Technology University, Noakhali, 3814 Bangladesh; 3 Department of Genetic Engineering and Biotechnology, University of Rajshahi, Rajshahi, 6205 Bangladesh

**Keywords:** Taro rhizosphere, *Enterobacter adelaidei*, Plant growth-promotion, Genomic analysis, Salinity stress

## Abstract

**Background:**

*Enterobacter adelaidei*, an extensively drug resistant strain, was recently described and isolated from hospital wastewater in Australia. However, its distribution in plant-associated environments has not been documented yet. This study presents a whole-genome-based characterization of *E. adelaidei* strain CELK12, isolated from the rhizosphere of taro (*Colocasia esculenta* L.) and its plant growth-promoting performance under saline stress conditions.

**Results:**

Genomic analyses confirmed the strain as *E. adelaidei* (ANI: 96.49%, dDDH: 71.90%). The genome of strain CELK12 (4.68 Mb, 53.5% GC content) was predicted to contain key genes involved in nutrient acquisition (*gcd*,* pqqC*,* amtB*), phytohormone biosynthesis (*ipdC*,* trpE*), iron transport (*entB*,* feoB*), and stress tolerance (*otsA*,* katG*,* sodA*,* oxyR*). These genomic features suggest the potential for rhizosphere competence and plant-beneficial activity of strain CELK12. Comparative genomics suggested a trend toward an open pan-genome among the selected strains (Heap’s law α = 0.11) and strain-specific divergences in secondary metabolite production, consistent with possible environmental adaptation. Genomic and phenotypic assessments revealed that while CELK12 carries certain chromosomally encoded resistance genes, it lacks plasmids, shows no hemolytic activity, and exhibits broad phenotypic susceptibility to major antibiotic classes. The seed germination and pot investigations confirmed significant enhancement of rice growth under saline conditions (100–150 mM NaCl) following CELK12 treatment.

**Conclusion:**

The findings of this study expand the known ecological range of *E. adelaidei* to include plant-associated niches and provide the first genomic predictions and experimental evidence of potential plant-beneficial traits within this specific lineage of the species.

**Supplementary Information:**

The online version contains supplementary material available at 10.1186/s12864-026-13081-3.

## Background

Plant growth-promoting rhizobacteria (PGPR) are critical drivers of sustainable agriculture, facilitating nutrient acquisition, stress tolerance, and pathogen suppression in crops [1, 2]. While PGPR diversity has been widely studied in major cereals and legumes, the rhizospheres of underexplored crops such as taro (*Colocasia esculenta*) remain largely neglected [3–10]. As a tropical tuber crop often grown in marginal or stress-prone habitats, taro represents a unique ecological niche. The unique composition of its root exudates, combined with fluctuating oxygen availability in the rhizosphere, fosters a robust microbial population equipped with specific adaptations for nutrient cycling and environmental stress tolerance [11, 12]. Consequently, these ecological characteristics make the taro rhizosphere a promising reservoir of functionally diverse and climate-adapted microbial lineages, with potential applications in developing sustainable agricultural solutions.

The genus *Enterobacter* comprises both clinically important and environmental species, with species displaying remarkable ecological versatility [13, 14]. Several *Enterobacter* species with plant growth-promoting attributes have been recovered from the rhizosphere of diverse crops, where they contribute to nutrient mobilization, phytohormone production, and stress alleviation [15–20]. However, the agricultural application of *Enterobacter* is often scrutinized due to the presence of various opportunistic human pathogens within the genus [21]. To address this, a robust biosafety framework integrating phenotypic assays with genomic screening for virulence factors and antibiotic resistance markers is indispensable [22, 23]. This is particularly important given the potential for high degree of genomic flexibility and the tendency for horizontal gene transfer observed within this genus, which may influence both environmental survival and clinical risk [24, 25].

The identification of *Enterobacter adelaidei* in the rhizosphere, moving beyond its initial classification as a clinical type strain (ECC3473) [26], represents a significant genomic shift that reveals a previously unrecognized capacity for metabolic versatility and environmental adaptability. However, given the close phylogenetic proximity between clinical and environmental *Enterobacter* lineages, conventional biochemical assay is often insufficient for functional differentiation [14, 27]. This proximity transforms *E. adelaidei* into a compelling model for comparative genomics to identify the specific “genomic fingerprints” of environmental resilience. Analyzing its genetic makeup is crucial for uncovering the evolutionary mechanisms that permit a lineage to shift from a potentially pathogenic background toward a beneficial, root-associated lifestyle. To fully understand the metabolic blueprints of new isolates, high-resolution comparative and pan-genomic evaluations are required. Such analyses establish a clear framework for differentiating the functional innovations required for rhizosphere colonization from traits that pose a clinical threat [28, 29].

In this study, we describe the isolation and genome-based characterization of strain CELK12 from the taro rhizosphere, representing the first documented case of a plant-associated lineage within *E. adelaidei*. We hypothesized that the adaptation of *E. adelaidei* to the rhizosphere niche is driven by a distinct accessory genome enriched with genes for environmental monitoring, stress resilience, and plant-microbe signaling, alongside a relative reduction of clinical virulence determinants. We evaluated this by using comparative and pan-genomic approaches to determine the divergence in features associated with nutrient acquisition, stress resilience, and root colonization. By comparing the genomic variations between the strain CELK12 and the clinical type strain, we aimed to define the genetic hallmarks of ecological specialization and biosafety. The functional importance of these genetic markers was further validated through in vivo trials, where rice inoculation under saline conditions displayed the strain’s ability to enhance plant growth. Ultimately, this research broadens our insight into the evolutionary trajectory of *E. adelaidei*, emphasizing the importance of exploring less-studied crop rhizosphere habitats to uncover the genomic mechanisms and metabolic innovations that drive niche-specific specialization.

## Materials and methods

### Isolation, screening and functional characterization of rhizobacteria

Twenty-three rhizosphere soil samples were collected from healthy taro (*Colocasia esculenta* L.) plants grown in five districts of Bangladesh: Rajshahi, Rangpur, Khulna, Noakhali, and Lakshmipur. The samples were transported under cold-chain conditions, and stored at 4 °C until processing. Soil pH was recorded, and bacteria were isolated by serial dilution plating (10⁻¹ to 10⁻^6^) on nutrient agar (NA) followed by incubation at 28 ± 2 °C for 48 h. Morphologically distinct colonies were purified by repeated streaking. All isolates were initially screened for phosphate solubilization on Pikovskaya’s agar [30], and positive strains were further evaluated for multiple plant growth-promoting traits, including phosphate and potassium solubilization, indole-3-acetic acid production, nitrogen fixation, siderophore, protease, ammonia, hydrogen cyanide production, and biofilm formation using previously reported standard protocols [31–39]. Abiotic stress tolerance of the rhizobacterial isolates was assessed in Luria-Bertani (LB) broth under drought (PEG 6000, 5–25%), salinity (NaCl, 2.5–10%), temperature (30–50 °C), and pH (4–12) stresses as described earlier [40, 41]. Optical density (OD) at 600 nm was obtained using a spectrophotometer and OD_600_ ≥ 0.1 was considered positive growth [42]. Antagonistic activity of the isolates was further evaluated against *Fusarium fujikuroi* (MT856372) and *Fusarium concentricum* (MT856371) using the dual culture assay on Potato Dextrose Agar (PDA), with percent inhibition calculated relative to controls [39].

### Morphological, biochemical and molecular identification of selected rhizobacteria

For the selected PGPR isolate, morphological, biochemical and molecular identification were performed. Determination of colony morphology (size, shape, margin, elevation, texture, opacity, color and odor), Gram reaction, and standard biochemical tests including catalase, oxidase, motility, triple sugar iron, indole, Methyl Red-Voges Proskauer (MR-VP), citrate, urease and nitrate reduction were performed following established protocols, with preliminary identification based on Bergey’s Manual [37, 43]. Genomic DNA was extracted using the Monarch^®^ genomic DNA kit, and the 16S rRNA gene was amplified using universal primers 27 F and 1492R. PCR products were visualized on 1% agarose gels, purified and sequenced. The resulting nucleotide sequences were edited in BioEdit v7.7.1 [44] and compared with GenBank entries using BLAST. Partial 16S rRNA gene sequences were deposited in GenBank. Phylogenetic analysis was conducted using reference sequences from GenBank, with *Escherichia coli* as an outgroup, multiple sequence alignment was carried out by ClustalW [45], and the tree was constructed in MEGA 11 [46] using the Neighbor-Joining method [47] with 1,000 bootstrap replications.

### Whole-genome sequencing, assembly and functional annotation

Whole-genome sequencing was performed at the International Centre for Diarrhoeal Disease Research, Bangladesh Genome Centre (iGC), Dhaka, using the Illumina NextSeq 2000 platform. The paired-end raw FASTQ files were uploaded to the Galaxy server, where sequence quality was assessed using FastQC v0.12.1 [48], followed by quality filtering and trimming with FastP v1.0.1 [49]. De novo genome assembly was conducted using SPAdes v3.15.5 [50], and the assembly quality was evaluated using QUAST v5.3.0 [51]. Genome completeness and contamination were additionally assessed using CheckM v1.2.4 [52]. The resulting assembly reached a mean sequencing coverage of 81.23× and high-quality standards, characterized by 95 contigs, an N50 of 701,616 bp, and a GC content of 53.5%. CheckM analysis confirmed a genome completeness of 99.07% with a low contamination rate of 1.11%, meeting the criteria for high-quality draft genomes. Genome annotation and functional prediction were carried out using Prokka v1.14.6 [53] and Rapid Annotation using Subsystem Technology (RAST) web server [54]. Ribosomal RNA gene operons were additionally identified using barrnap v0.9 [55] with default parameters to resolve possible overestimation of rRNA genes generated due to the draft genome assembly. Circular genome visualization was generated using the Proksee server [56]. The assembled genome sequence was deposited in GenBank under BioProject accession PRJNA1344764 and BioSample accession SAMN54939169.

### Phylogenetic analysis of strain CELK12

The whole-genome sequence of strain CELK12 was analyzed using the Type Strain Genome Server (TYGS) to determine its phylogenetic placement [57]. Phylogenetic reconstruction was performed using FastME v2.1.6.1 [58] based on Genome BLAST Distance Phylogeny (GBDP) distances derived from whole-genome sequences. Branch lengths were expressed according to the GBDP distance formula *d*_*5*_, and the resulting tree was midpoint rooted [59]. Digital DNA-DNA hybridization (dDDH) analysis was performed to evaluate genome-level relatedness between strain CELK12 and the selected type strains using the Genome-to-Genome Distance Calculator (GGDC) tool integrated into TYGS [60]. A cut-off value of 70% dDDH was considered indicative of species-level affiliation, in accordance with established taxonomic standards [61]. Average nucleotide identity (ANI) was determined using the JSpeciesWS web server, which calculates genome-wide nucleotide identity based on pairwise comparisons of whole-genome sequences [62]. A cut-off value of ≥ 95–96% ANI was applied for species assignment [63, 64].

### Genome-based prediction of plant growth-promoting traits and secondary metabolite biosynthetic gene clusters

Plant growth-promoting traits (PGPTs) were predicted using the PLaBAse v1.02 PGPT-Pred module employing the BLASTP+HMMER aligner/mapper for gene prediction [65, 66]. To quantify plant-microbe interaction-related genes in the genome, annotated protein sequences from bacterial isolate were analyzed using the PIFAR-Pred pipeline in PLaBAse. A combined BLASTP+HMMER approach was employed to identify genetic determinants associated with plant-microbe interaction. Secondary metabolite biosynthetic gene clusters (BGCs) were identified and annotated using antiSMASH v8.0.4 [67, 68], and predicted clusters were compared with curated reference BGCs in the MIBiG database.

### Comparative genomic characterization of *E. adelaidei* CELK12 and the type strain ECC3473

Comparative genomic analysis of CELK12 was conducted against the only available type strain of the species, the clinical isolate *E. adelaidei* ECC3473 (GenBank accession: CP154353). Both genomes were functionally annotated using eggNOG-mapper v2.1.8 [69] in Galaxy platform to assign COG [70], KEGG [71], and CAZy [72] classifications for comparative assessment. PGPTs and BGCs were compared based on PGPT-Pred and antiSMASH outputs, respectively [65, 68]. To compare biosafety-related features, the genomes were screened for antimicrobial resistance and virulence-associated genes using ABRicate v1.0.1 [73] on the Galaxy platform against the Resfinder [74], Comprehensive Antibiotic Resistance Database (CARD) [75] and the Virulence Factor Database (VFDB) [76] based on minimum thresholds of ≥ 80% sequence identity and ≥ 80% coverage, and hits below these cutoffs were not considered. Presence-absence patterns of functional genes and genomic determinants were subsequently analyzed to identify shared and strain-specific features between the two genomes.

### Comparative pan-genome analysis of selected plant-associated and clinical *Enterobacter* strains

Because *E. adelaidei* is a recently described species with only one publicly available reference genome (ECC3473), a comparative pan-genome analysis of selected *Enterobacter* strains was performed to contextualize CELK12 within a broader evolutionary and functional framework. Eight plant-associated and seven clinical or antimicrobial-resistant *Enterobacter* strains were selected from the NCBI RefSeq database, prioritizing type and high-quality genomes while maintaining CELK12 as the focal strain. To minimize species-level bias, no more than two genomes per species were included, and plant-associated and clinical isolates were paired where possible. All genome assemblies were quality-checked and uniformly re-annotated using Prokka v1.14.6 [53] to ensure consistency in downstream analyses. Pan-genome reconstruction was conducted using Roary v3.13.0 [77] on the Galaxy platform. Pan-genome trends were evaluated using Heaps’ law [78]; however, given the limited number of genomes analyzed, these estimates were used for comparative purposes only. Functional annotation of gene clusters was performed based on COG classifications [70].

### Rhizobacterial inoculation and plant growth promotion under salinity stress

Effects of CELK12 inoculation on plant growth under salinity were evaluated using rice (*Oryza sativa* L., variety: BRRI Dhan 28) through seed germination and pot experiments. For germination tests, surface-sterilized seeds (70% ethanol, 2% sodium hypochlorite) were rinsed, air-dried, and primed with bacterial suspensions (10⁸ CFU/mL) for 30 min, whereas controls received only sterile water [79]. Treated seeds (20 per treatment) were incubated on moistened sterile filter paper in Petri dishes at 28 ± 2 °C under a 12 h photoperiod and exposed to 50, 100, 150, or 200 mM NaCl (5, 9, 14.8, and 18 dSm^− 1^). Germination was recorded based on radicle emergence and expressed as percentage germination [80]. For pot experiments, five-days-old seedlings were transplanted into double-sterilized sandy loam soil (200 g per pot) and inoculated with 20 mL bacterial suspension at 1 and 7 days after transplantation; uninoculated plants served as controls [81]. Salinity stress was applied after the second inoculation by irrigation with 100 or 150 mM NaCl at 2-days intervals. Plants were kept under controlled conditions (26 ± 2 °C, 16/8 h light/dark cycle) with three biological replicates per treatment. After three weeks of planting, root and shoot lengths were measured, and fresh and dry biomass were determined following oven-drying at 70 °C for 48 h.

### Preliminary biosafety evaluation for environmental PGPR application

Preliminary biosafety assessment of *E. adelaidei* CELK12 for environmental PGPR application was conducted through antibiotic susceptibility and hemolysis assays. Antibiotic susceptibility was tested using the disk diffusion method [82] against ten antibiotics: piperacillin-tazobactam (TZP, 110 µg), ceftazidime (CAZ, 30 µg), cefepime (CPM, 30 µg), aztreonam (ATM, 30 µg), gentamicin (CN, 10 µg), amikacin (AK, 30 µg), tobramycin (TOB, 10 µg), ciprofloxacin (CIP, 5 µg), imipenem (IMP, 10 µg), and meropenem (MEM, 10 µg). Hemolytic activity was evaluated on blood agar supplemented with 5% defibrinated sheep blood following incubation at 28 ± 2 °C for 24–48 h [83]. Hemolysis patterns were determined as α-hemolysis (greenish discoloration), β-hemolysis (clear zone), or γ-hemolysis (no hemolysis), with γ-hemolysis considered non-hemolytic and environmentally safe.

### Statistical analysis

To ensure reproducibility, all the experiments were conducted independently in triplicate and statistical analysis were manifested using SPSS statistical package v20.0. All data are expressed as Mean ± Standard Deviation (SD) to reflect the observed biological variability. Statistical significance was determined using one-way Analysis of Variance (ANOVA), followed by Duncan’s Multiple Range Test (DMRT) at a *P* ≤ 0.05 significance level to identify distinct treatment groups.

## Results

### Isolation and functional characterization of CELK12

**Fig. 1:**
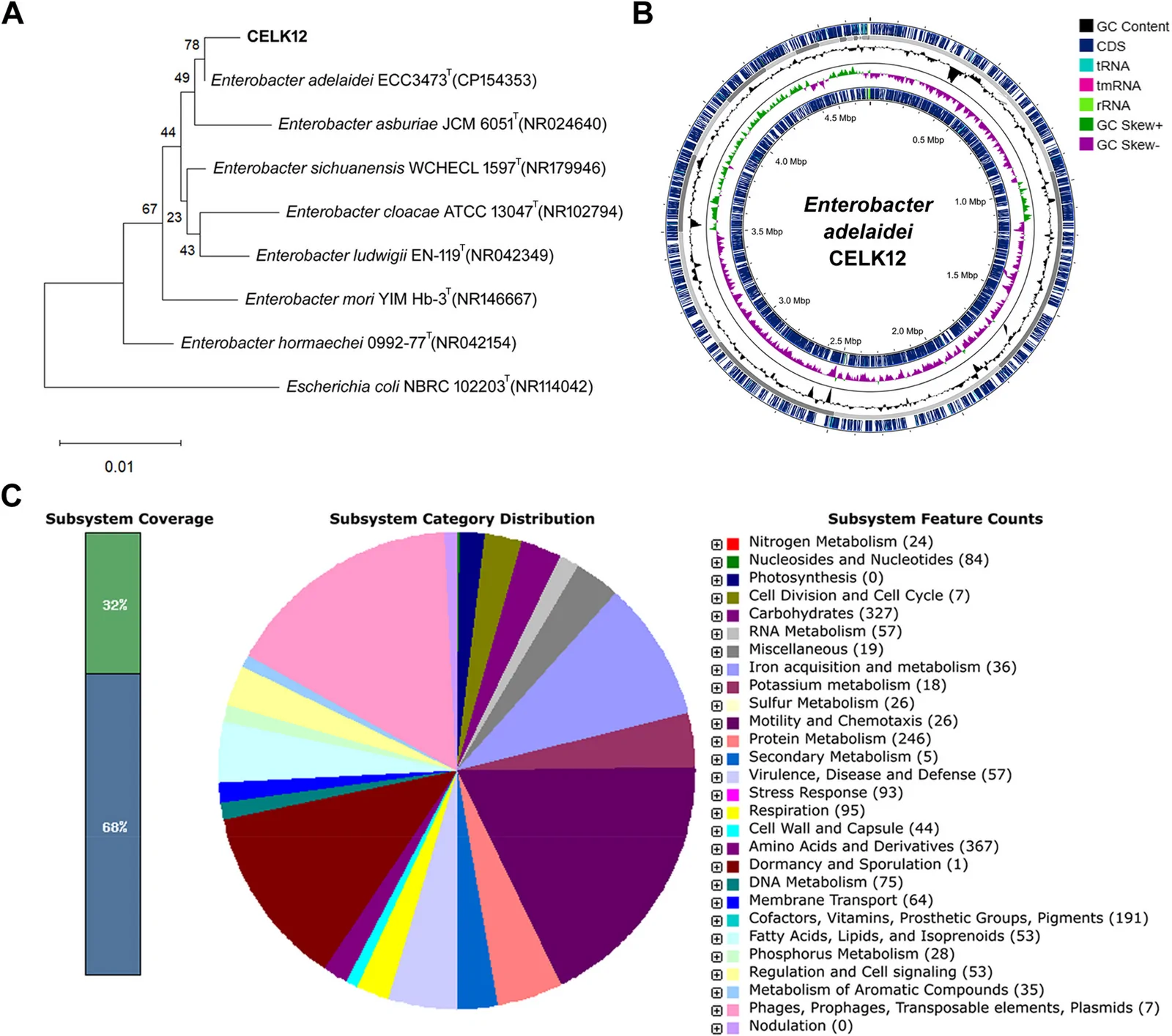
**A** Phylogenetic analysis of strain CELK12 based on 16S rRNA gene sequences. **B** Circular representation of the CELK12 genome. **C** Functional annotation and subsystem classification generated using RAST

A total of 202 rhizobacterial isolates were obtained from twenty-three taro rhizosphere soil samples (Supplementary Table S1). Twenty-eight isolates (13.9%) showed phosphate-solubilizing activity with phosphate solubilization index ranging from 1.08 to 2.17 and exhibited marked variability in other PGP traits (Supplementary Table S2). Based on PGP activities, twenty isolates were further subjected to abiotic stress tolerance and antifungal assay (Table 1, Supplementary Table S3-S4). Among the isolates, CELK12 was prioritized for whole-genome sequencing due to its balanced combination of plant growth-promoting traits including phosphate (PSI 1.21) and potassium solubilization, indole-3-acetic acid production (59.92 µg/mL), siderophore and ammonia production, and strong biofilm formation (Table 1, Supplementary Fig. S1). Notably, the isolate tolerated multiple abiotic stresses, surviving drought (20% PEG 6000), high salinity (7.5% NaCl), elevated temperature (40 °C), and a broad pH range (4–10). In addition, CELK12 significantly inhibited fungal pathogen *Fusarium concentricum* (47.83% inhibition). Morphological, biochemical and 16S rRNA gene sequence-based analysis identified the isolate CELK12 as *Enterobacter* sp. (GenBank accession no. PX878863) (Fig. 1A, Supplementary Table S5).

**Table 1:** In vitro characterization of rhizobacterial isolates from the taro rhizosphere for plant growth-promoting traits, tolerance to abiotic stress conditions, and antifungal activity

Isolate code	PGP traits									Abiotic stress tolerance				Antifungal traits (% inhibition)	
	P solubilization index	K solubilization	IAA production (µg/mL)	Nitrogen fixation	Siderophore	Protease	Ammonia	HCN	Biofilm	Drought (% PEG 6000)	Salt (% NaCl)	Temperature (°C)	pH	*F. fujikuroi*	*F. concentricum*
CERJ1	2.00 ± 0.14hi	-	122.67 ± 0.98n	-	+	-	+	-	+	15	2.5	45	6–12	-	-
CERN2	1.31 ± 0.05a-g	+	79.74 ± 1.67k	-	-	-	+	-	+	25	7.5	45	6–10	-	45.05
CEKH2	1.57 ± 0.08 g	+	69.68 ± 0.80i	-	-	-	+	-	+	15	5	45	4–10	-	-
CENK1	1.46 ± 0.10d-g	+	29.45 ± 0.44f	-	+	+	+	+	+	20	2.5	40	6–10	-	45.05
CENK2	1.39 ± 0.10b-g	+	27.46 ± 0.66e	-	+	+	+	+	+	20	2.5	45	6–10	-	-
CENK3	1.48 ± 0.03efg	+	-	-	+	-	+	-	+	20	7.5	45	4–10	53.19	-
CENK4	1.89 ± 0.19 h	+	39.10 ± 1.23 g	-	+	+	+	+	+	20	5	45	4–10	-	-
CENK5	1.94 ± 0.34hi	+	8.51 ± 0.73b	+	+	-	+	-	+	25	5	40	6–10	-	-
CENK6	1.36 ± 0.13a-g	+	-	+	+	-	+	-	+	20	2.5	45	6–10	-	-
CENK7	1.09 ± 0.00a	+	20.68 ± 0.73d	+	+	+	+	+	+	20	5	40	6–10	-	-
CENK8	1.19 ± 0.01a-d	+	2.14 ± 0.98a	-	+	-	+	-	+	20	10	45	4–10	-	43.66
CELK2	1.97 ± 0.26hi	+	-	+	+	+	+	-	-	25	7.5	45	6–10	72.35	70.41
CELK4	2.08 ± 0.38hi	+	-	+	+	+	+	+	-	25	5	45	6–10	70.23	78.86
CELK5	1.21 ± 0.01a-e	+	27.05 ± 1.10e	+	+	+	+	+	+	25	5	45	4–10	-	-
CELK6	1.24 ± 0.02a-f	+	7.87 ± 0.87b	+	+	-	+	-	+	20	5	45	6–10	-	-
CELK8	2.17 ± 0.07i	+	117.11 ± 1.07 m	+	+	-	+	-	+	20	7.5	45	6–10	-	46.50
CELK9	1.52 ± 0.04 fg	+	2.14 ± 0.80a	+	+	-	+	-	+	25	5	45	6–10	-	45.05
CELK10	1.37 ± 0.11a-g	+	-	+	+	-	+	-	+	20	7.5	45	6–10	-	38.04
CELK11	1.14 ± 0.07abc	+	-	+	+	-	+	-	+	20	5	45	6–10	-	46.44
CELK12	1.21 ± 0.01a-e	+	59.92 ± 0.73 h	-	+	-	+	-	+	20	7.5	40	4–10	-	47.83

+ denotes positive activity, - denotes negative activity, the numerical values are showed as the means ± standard deviations (SDs) of triplicates. Different lowercase letters within a column denote statistically significant differences as per DMRT (*P* ≤ 0.05)

### Genomic properties of strain CELK12

The whole-genome sequence of isolate CELK12 was analyzed and deposited at GenBank under the accession JBUBKU000000000. A circular genomic map of the isolate was generated using Proksee (Fig. 1B). The genome size of CELK12 was 4,689,336 bp with a GC content of 53.5%. Altogether, strain CELK12 comprises 4,547 genes, including 4,348 protein-coding genes, 65 pseudogenes, 0 complete and 2 partial (fragmented assembly; multiple 5 S fragments) rRNA operons, 82 tRNAs, 7 ncRNAs and no plasmid (Table 2).

**Table 2:** General genomic characteristics of strain CELK12

Characteristics	*E. adelaidei* CELK12
Genome size (bp)	4,689,336
GC content (%)	53.5
Contigs	95
N50 value (bp)	701,616
L50 value	3
Completeness (%)	99.07
Contamination (%)	1.11
Coverage	81.23×
Total genes	4,547
Protein-coding genes (CDS)	4,348
tRNA	82
rRNA operons	0 complete, 2 partial
ncRNAs	7
Pseudogenes	65
Plasmid	0

RAST subsystem annotation of the CELK12 genome assigned 68% of predicted coding sequences to functional subsystems, while 32% remained unclassified (Fig. 1C). The functional profile was dominated by core metabolic categories, particularly amino acids and derivatives (367 features), carbohydrates (327), protein metabolism (246), and cofactors, vitamins, prosthetic groups, and pigments (191), indicating substantial metabolic versatility. Genes related to environmental adaptation and cellular maintenance were also prominent, including stress response (93 features), respiration (95), membrane transport (64), DNA metabolism (75), and cell wall and capsule formation (44). Subsystems associated with rhizosphere competence and plant-associated functions were identified, such as phosphorus metabolism (28 features), iron acquisition and metabolism (36), nitrogen metabolism (24), motility and chemotaxis (26), and metabolism of aromatic compounds (35). Additionally, genes related to virulence, disease and defense (57 features) and mobile genetic elements (7 features) were detected, whereas photosynthesis and nodulation subsystems were absent.

### Phylogenomic placement and taxonomic confirmation of CELK12

**Fig. 2:**
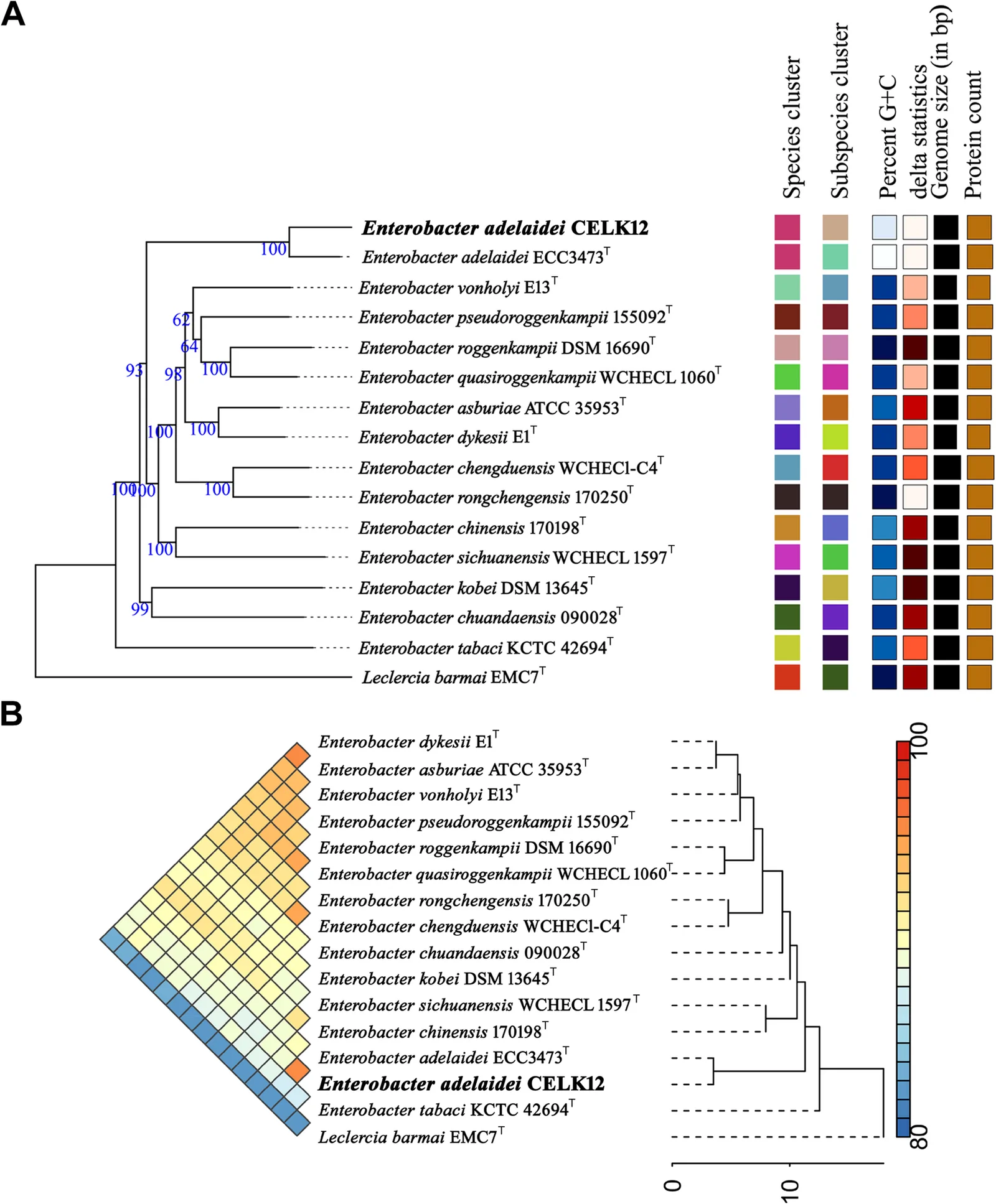
**A** Genome BLAST distance phylogeny (GBDP) tree. The numbers above the branches are GBDP pseudo-bootstrap support values > 60% from 100 replications, with an average branch support of 92.0%. **B** Comparison of the average nucleotide identity (ANI) values between each genome of the 16 strains of *Enterobacter* spp. generated using Integrated Prokaryotes Genome and pan-genome Analysis (IPGA) web server

Whole-genome-based phylogenetic analysis using TYGS, together with ANI and dDDH values, clearly assigned strain CELK12 to the species *E. adelaidei* (Fig. 2 and Supplementary Table S6-S7). The strain showed ANI and dDDH values (96.49% and 71.90% respectively) above the accepted species delineation thresholds (ANI ≥ 95% and dDDH > 70%) when compared with the type strain *E. adelaidei* ECC3473, confirming its taxonomic placement.

### Plant growth-promoting traits and microbe-plant interaction factors in *E. adelaidei* CELK12

**Fig. 3:**
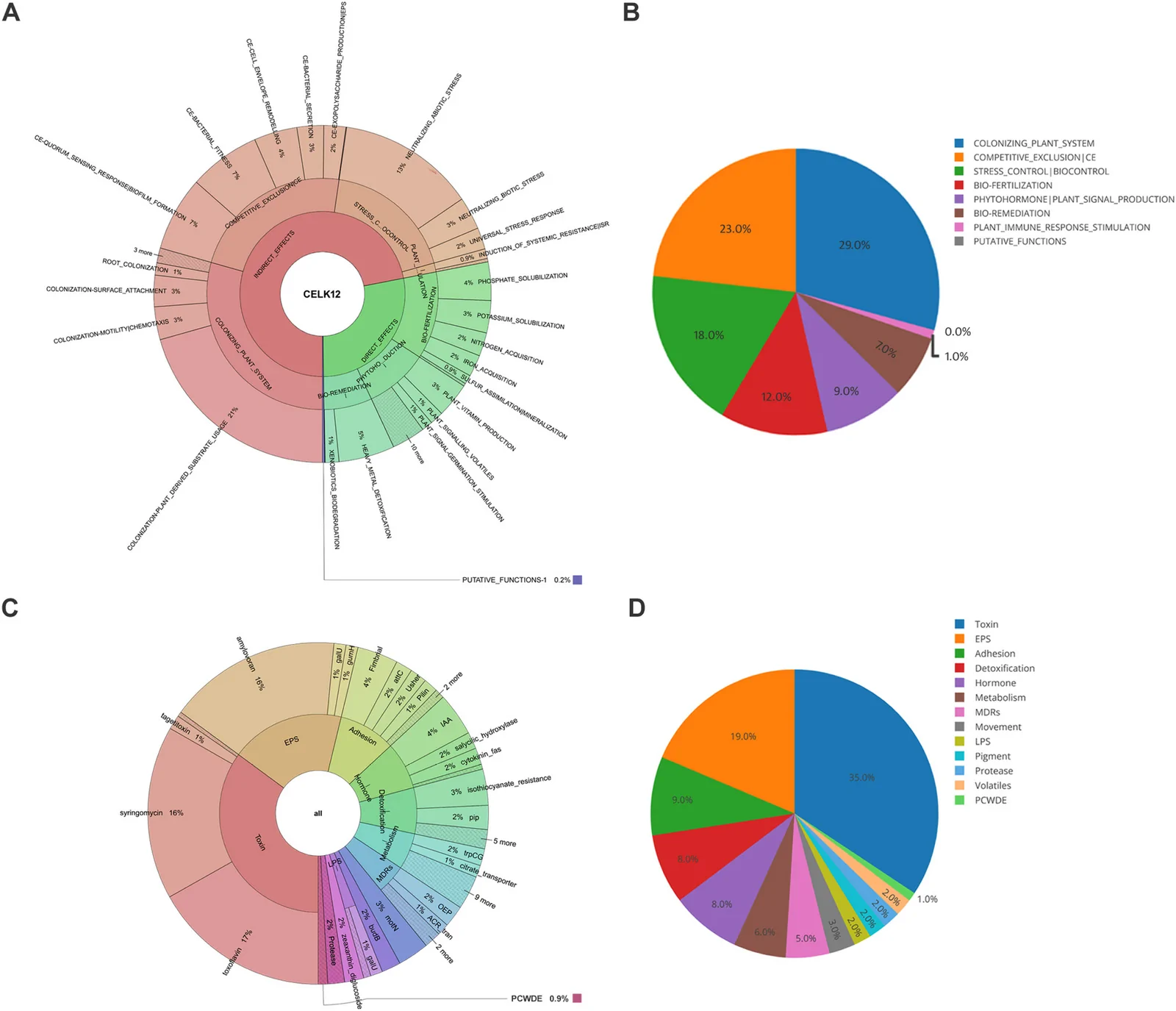
**A** Krona visualization showing the major plant growth-promoting traits identified in the CELK12 genome. **B** Pie chart depicting the genomic distribution of plant growth-promoting trait (PGPT) genes. **C** Krona plot illustrating proportion of genetic determinants associated with plant-bacteria interactions detected in strain CELK12, **D** Pie chart showing the relative abundance of genes involved in plant-bacteria interaction processes within the genome

Genome mining of CELK12 using PLaBAse identified diverse plant growth-promoting traits (PGPT) and microbe-plant interaction-associated genetic determinants (Fig. 3A-B). PGPT annotation showed that colonization-related functions were predominant, including plant defense stimulation (21%), biofilm formation (7%), quorum sensing and competitive exclusion (4–5%), and motility or surface attachment-mediated root colonization (1–3%). Stress tolerance-associated genes comprised 15% of the PGPT repertoire, including abiotic stress response, detoxification, and induction of systemic resistance. Direct growth promotion traits included phosphate solubilization (4%), potassium solubilization (3%), nitrogen acquisition (2%), and mineral nutrient assimilation. Additional functions related to phytohormone production, vitamin biosynthesis, and signaling were detected at lower frequencies. Overall, PGPT categories were dominated by colonizing plant system functions (29%), followed by competitive exclusion (23%), stress control/biocontrol (18%), biofertilization (12%), phytohormone production (9%), bio-remediation (7%), and plant immune response stimulation (1%), with minimal putative functions.

Prediction of plant-microbe interaction factors revealed that toxin-related determinants were the largest category (35%), followed by exopolysaccharide biosynthesis (19%) and adhesion-associated functions (9%). Additional categories included detoxification (8%), hormone-related functions (8%), and metabolism-associated interaction factors (6%) (Fig. 3C-D). Multidrug resistance systems (5%), movement-related traits, lipopolysaccharide biosynthesis, pigment production, proteases, and volatile-associated functions were present at lower proportions (2–3%), whereas plant cell wall-degrading enzymes represented a minor fraction (1%). Complete genome annotations generated using PGPT-Pred and PIFAR-Pred are presented in Supplementary Tables S8-S9.

### Secondary metabolites biosynthetic gene cluster (BGC) analysis of *E. adelaidei* CELK12

Genome mining of *E. adelaidei* CELK12 identified multiple biosynthetic gene clusters spanning diverse metabolite classes, including azole-containing ribosomally synthesized and post-translationally modified peptides, non-ribosomal peptide synthetase-independent siderophores, NRPS-associated metallophores, terpene precursors, and arylpolyene clusters (Table 3, Supplementary Fig. S2). Several clusters showed high similarity to known biosynthetic systems, notably frederiksenibactin-like and arylpolyene clusters, while others displayed medium similarity to aerobactin or lacked close homologs in reference databases. The presence of both conserved and divergent clusters indicates a heterogeneous secondary metabolite repertoire encoded within the CELK12 genome.

**Table 3:** Secondary metabolite biosynthetic gene clusters (BGCs) of CELK12 identified using AntiSMASH

Region	BCG type	Location	Most similar known cluster	Similarity
1.1	azole-containing-RiPP	622,880 − 649,302	-	-
2.1	NI-siderophore	815,742–837,975	aerobactin	Medium
6.1	terpene-precursor	107,758 − 128,657	-	-
6.2	NRP-metallophore, NRPS	263,840 − 311,213	frederiksenibactin	High
8.1	arylpolyene	74,922 − 118,515	aryl polyenes	High

azole−containing−RiPP azole−containing−Ribosomally synthesized and Post−translationally modified Peptide, NI−siderophore Non−Ribosomal Peptide Synthetase−independent Siderophore, NRP− metallophore Non−Ribosomal Peptide− metallophore, NRPS Non−Ribosomal Peptide Synthetase

### Comparative genomic analysis of *E. adelaidei* CELK12 with *E. adelaidei* ECC3473

Comparative genomic analysis between *E. adelaidei* CELK12 and the reference strain ECC3473 revealed high overall functional conservation alongside strain-specific variation in genome organization, secondary metabolism, and resistance-associated features. Genome comparison showed that CELK12 possesses a smaller genome with fewer total genes, coding sequences, and pseudogenes than ECC3473, while maintaining comparable GC content and high genome completeness (Supplementary Table S10). In addition, CELK12 lacked plasmids and harbored a higher number of rRNA genes, highlighting distinct genomic architecture and potential differences in functional capacity between the strains.

COG- and KEGG-based functional annotation showed highly similar distributions of gene categories in both genomes (Supplementary Fig. S3). Genes related to transcription (K), translation and ribosome biogenesis (J), cell wall and membrane biogenesis (M), and energy production and conversion (C) predominated in both strains, indicating conservation of core cellular functions. Other functional groups, including carbohydrate transport and metabolism (G), amino acid transport and metabolism (E), signal transduction (T), and intracellular trafficking and secretion (U), were comparable or slightly enriched in ECC3473. KEGG pathway profiles showed overlapping distributions dominated by metabolism, genetic information processing, and cellular processes, with relatively higher gene counts in ECC3473 for several metabolic pathways and categories associated with human diseases and drug resistance. Comparative CAZy annotation indicated similar distributions of glycoside hydrolases and glycosyltransferases, with no enrichment of plant cell wall-degrading enzymes (Supplementary Fig. S4).

**Fig. 4:**
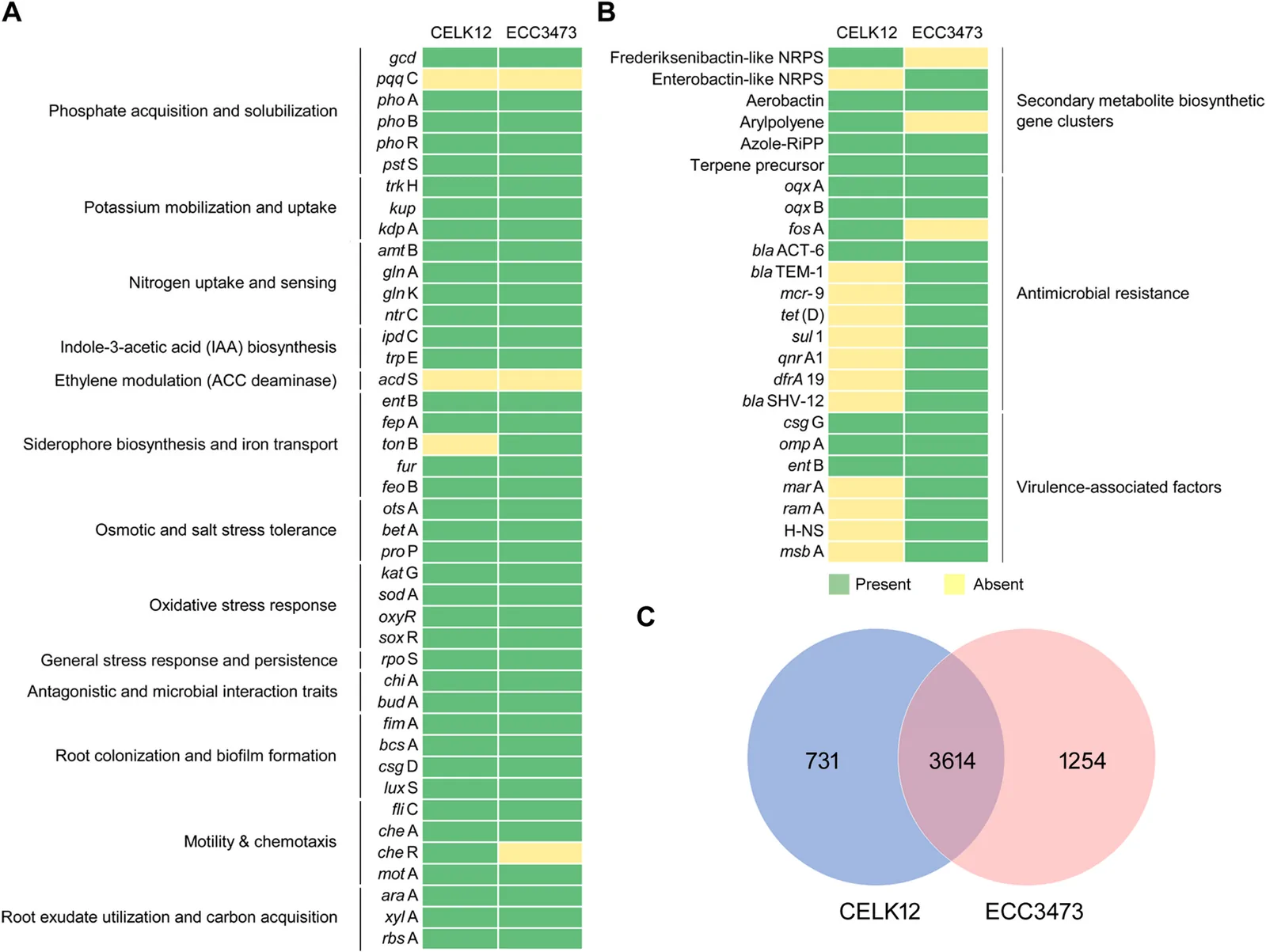
Comparative genomic analysis of CELK12 and the reference strain ECC3473. **A** Heatmap depicting the presence and absence of genes associated with plant growth-promoting traits, **B** secondary metabolite biosynthetic gene clusters (BGCs), antimicrobial resistance, and virulence factors. **C** Venn diagram illustrating the number of shared and strain-specific genes between CELK12 and ECC3473

Both genomes shared a conserved set of genes associated with environmental adaptation and plant-associated functions (Fig. 4A), including phosphate acquisition and regulation (*gcd*,* phoA*,* phoB*,* phoR*,* pstS*), potassium uptake (*trkH*,* kup*,* kdpA*), nitrogen assimilation and sensing (*amtB*,* glnA*,* glnK*,* ntrC*), indole-3-acetic acid biosynthesis (*ipdC*,* trpE*), and siderophore biosynthesis and iron transport (*entB*,* fepA*,* feoB*,* fur*), with variation in specific transport components (e.g., *tonB*). Both genomes additionally encoded genes associated with osmotic and oxidative stress tolerance (*otsA*,* betA*,* proP*,* katG*,* sodA*,* oxyR*,* soxR*), general stress response (*rpoS*), motility and chemotaxis (*fliC*,* cheA*,* motA*), root colonization and biofilm formation (*fimA*,* bcsA*,* csgD*,* luxS*), and utilization of plant-derived carbon sources (*araA*,* xylA*,* rbsA*).

Secondary metabolite analysis identified a conserved core of biosynthetic gene clusters (BGCs) in both strains, including aerobactin-type siderophore, azole-containing RiPP, and terpene precursor clusters (Fig. 4B). However, CELK12 encoded a high-confidence frederiksenibactin-like NRPS metallophore cluster, whereas ECC3473 carried an enterobactin-like NRPS cluster. An arylpolyene biosynthetic cluster was detected exclusively in CELK12.

Antimicrobial resistance screening showed that CELK12 contained a limited set of resistance genes (*oqxA*,* oqxB*,* fosA*,* blaACT-6*), whereas ECC3473 encoded a broader repertoire, including β-lactamases (*blaTEM-1*,* blaSHV-12*), colistin (*mcr-9*), tetracycline (*tetD*), sulfonamide (*sul1*), quinolone (*qnrA1*), and trimethoprim resistance (*dfrA19*) (Fig. 4B). Virulence-associated and global regulatory factors (*marA*,* ramA*, H-NS, *msbA*) were detected only in ECC3473, while both strains shared structural and surface-associated components (*csgG*,* ompA*) (Fig. 4B).

Gene content comparison identified 3,614 shared genes, indicating a conserved core genome, whereas 1,254 and 731 genes were uniquely present in ECC3473 and CELK12, respectively, reflecting lineage-specific genomic features (Fig. 4C).

### Comparative pan-genome analysis of selected plant-associated and clinical *Enterobacter* strains

**Fig. 5:**
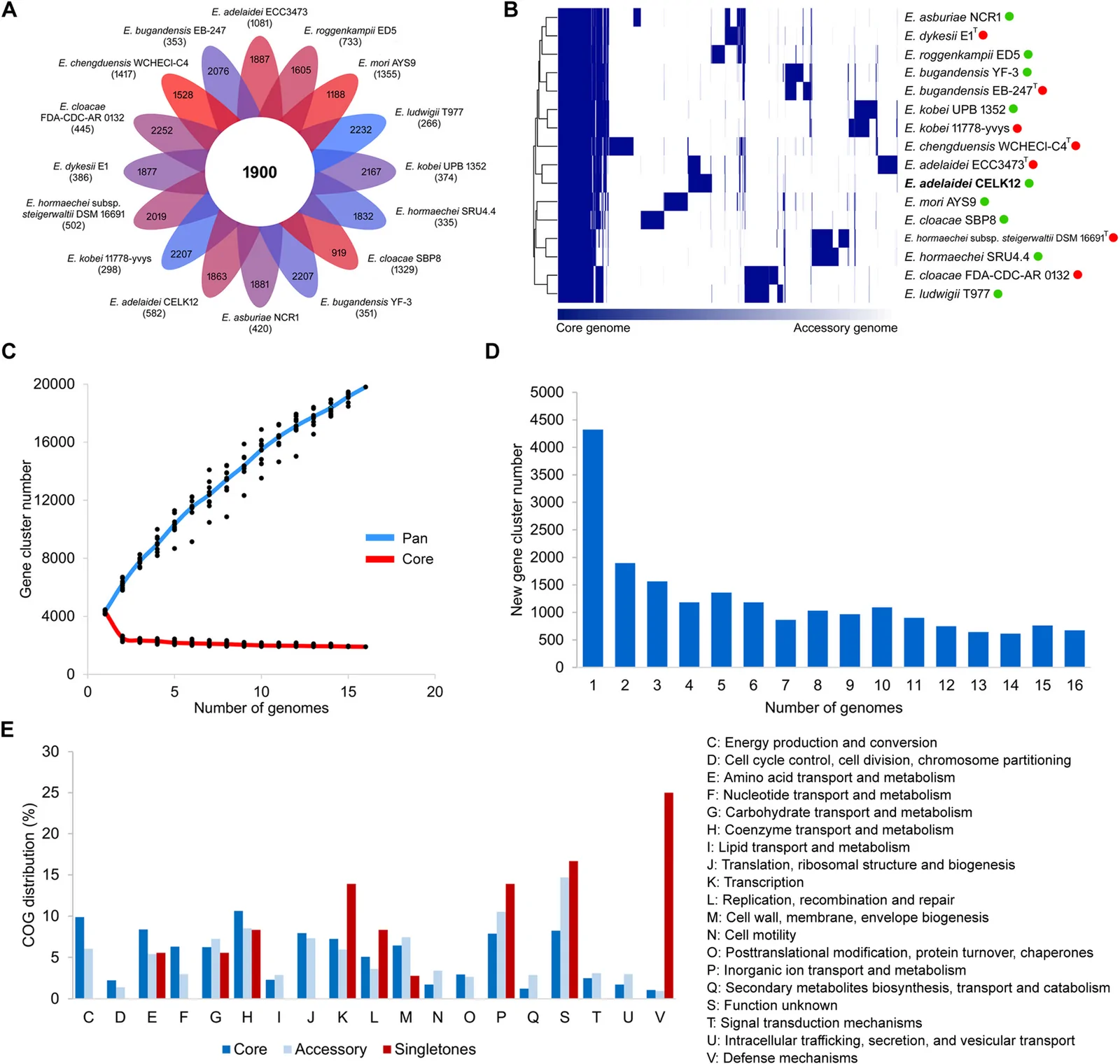
Genus-level pan-genome analysis of CELK12. **A** Flower plot illustrating the distribution of core, accessory, and strain-specific genes across the analyzed genomes. **B** Gene presence-absence matrix showing the core, accessory, and unique gene sets, accompanied by a maximum-likelihood phylogeny inferred from core genome sequences. Green dots indicate plant-associated strains, while the red dot represents the clinical strain. **C** Pan-genome and core genome accumulation curves, depicting genome expansion and core gene conservation with the addition of each genome. **D** Bar plot showing the number of novel genes identified with successive genome inclusion. **E** Functional categorization of core, accessory, and strain-specific genes based on COG annotations

A comparative pan-genome analysis of 16 selected *Enterobacter* genomes (Supplementary Table S11), including environmental, plant-associated, and clinical strains, identified a total of approximately 19,000 gene clusters, of which 1,900 were shared among all genomes and defined the core genome (Fig. 5A). Individual genomes contributed between 266 and 1,417 strain-specific gene clusters. Presence-absence based hierarchical clustering revealed distinct grouping patterns among the analyzed genomes (Fig. 5B). *E. adelaidei* CELK12 formed a separate branch from several clinical reference strains and clustered closer to plant-associated and environmental isolates based on accessory gene content. Pan-genome accumulation analysis showed a continuous increase in total gene clusters with the sequential addition of genomes, while the core genome size progressively decreased and approached stabilization (Fig. 5C). Heap’s law analysis yielded an α value of 0.11, suggesting a trend toward an open pan-genome; however, this observation should be interpreted cautiously given the limited number of genomes analyzed and is not intended to represent the entire genus. The number of newly introduced gene clusters remained high across genome additions, indicating an open pan-genome structure (Fig. 5D). COG-based functional classification indicated that core genes were mainly assigned to categories associated with translation, replication, energy production, and central metabolic processes (Fig. 5E). Accessory and singleton gene clusters were predominantly distributed among functional categories related to carbohydrate transport and metabolism, cell wall and membrane biogenesis, signal transduction, secondary metabolite biosynthesis, and defense mechanisms.

Annotation of the 582 singleton genes of *E. adelaidei* CELK12 showed that CELK12 possesses a gene repertoire biased toward environmental persistence and potential for rhizosphere adaptation rather than pathogenic specialization (Supplementary Table S12). The genes were primarily associated with motility and chemotaxis (*cheA/W/Y; flg*,* fli* operons), surface attachment and fimbrial assembly (*fimD/G*,* lpfA/B*,* sfmF/H*), and iron acquisition via siderophore biosynthesis and transport (*entB*,* entD*,* ybdZ*,* fepE*). Multiple stress-responsive genes, including peroxiredoxin (*bcp*), oxidative stress resistance factors, metal homeostasis proteins (*rcnB*,* zinT*), and mercury resistance regulators, indicate enhanced tolerance to variable soil conditions. Metabolic flexibility was supported by genes involved in citrate and glyoxylate metabolism (*citC/D*,* acnB*), nitrogen assimilation (*nrtA*,* norW*), and diverse carbohydrate-processing enzymes. Numerous HTH-type transcriptional regulators and cyclic di-GMP signaling components further suggest fine-scale regulatory control of motility and biofilm-associated traits. While genes related to efflux, defense, and genome plasticity were present, classical virulence determinants were not enriched, collectively supporting adaptation to a plant-associated ecological niche. Notably, 331 singleton genes were annotated as hypothetical proteins, suggesting the presence of previously uncharacterized or lineage-specific functions that may contribute to niche adaptation, ecological fitness, and unexplored plant-microbe interactions in the rhizosphere.

### Effect of *E. adelaidei* CELK12 inoculation on rice growth under salt stress

**Fig. 6:**
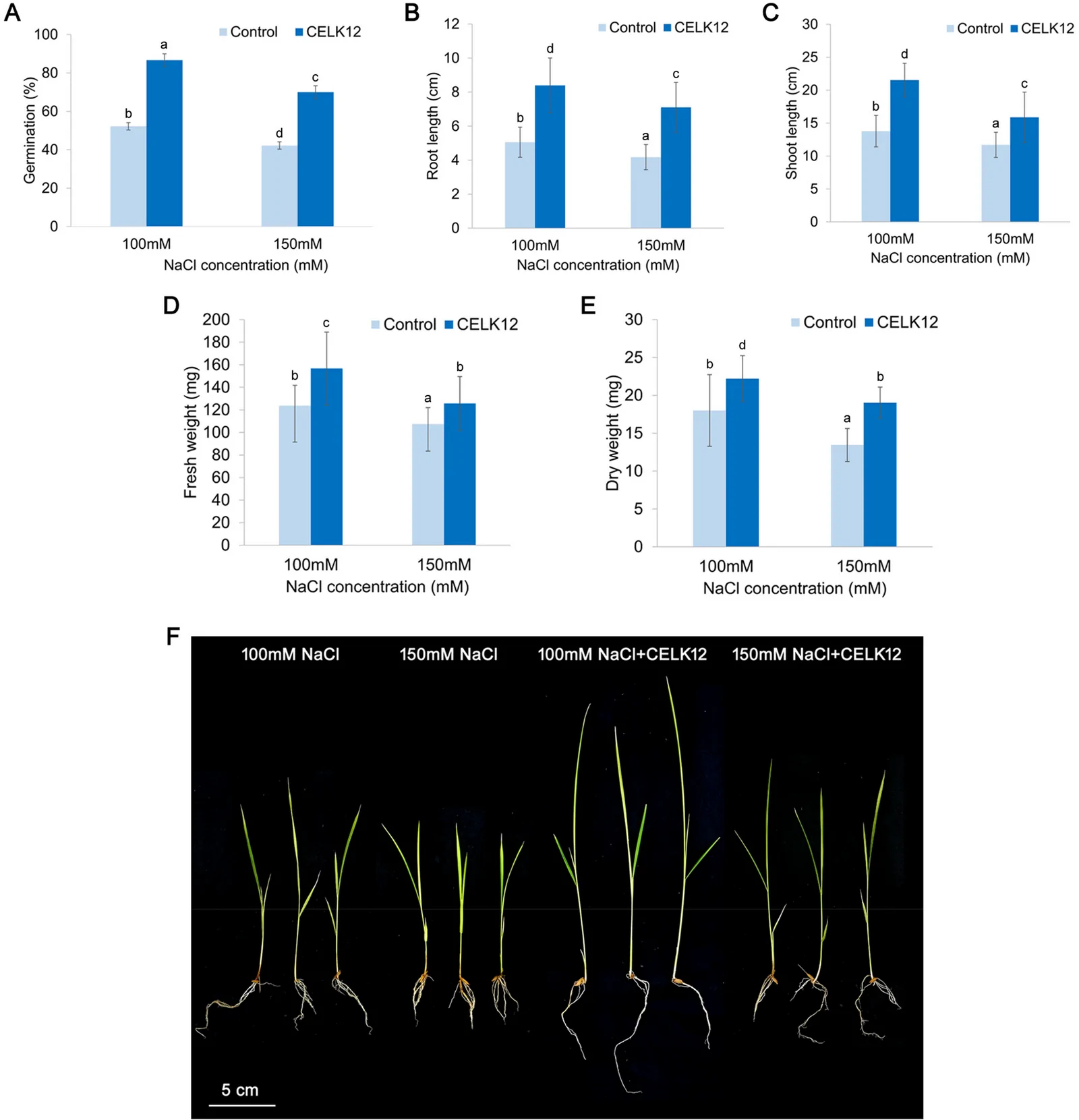
Effects of strain CELK12 inoculation on rice (*Oryza sativa* L.) seed germination and seedling growth under saline conditions (100 mM and 150 mM NaCl). Bar chart illustrating the enhancement in seed germination rate (**A**), root length (**B**), shoot length (**C**), fresh weight (**D**), and dry weight (**E**) relative to non-inoculated controls. (**F**) Qualitative image of root and shoot phenotype in response to100 mM and 150 mM NaCl stresses

Inoculation with *E. adelaidei* CELK12 markedly improved rice growth performance under saline conditions compared with uninoculated controls (Fig. 6A-F). Salt stress alone reduced germination and seedling growth, with germination decreasing from 52.22% at 100 mM NaCl to 42.22% at 150 mM NaCl, accompanied by reductions in root length (5.05 to 4.18 cm), shoot length (13.79 to 11.70 cm), fresh weight (123.68 to 107.28 mg), and dry weight (18.00 to 13.44 mg). In contrast, CELK12 inoculation significantly enhanced all measured parameters at both salinity levels (DMRT, *P* ≤ 0.05). At 100 mM NaCl, CELK12 treatment increased germination to 86.67%, root length to 8.39 cm, and shoot length to 21.53 cm, with corresponding increases in fresh (156.72 mg) and dry biomass (22.2 mg). Similarly, under 150 mM NaCl stress, inoculated plants exhibited higher germination (70.00%), root length (7.10 cm), shoot length (15.88 cm), fresh weight (125.68 mg), and dry weight (19.04 mg) compared with non-inoculated plants.

### Antibiotic susceptibility and hemolysis profile of *E. adelaidei* CELK12

**Fig. 7:**
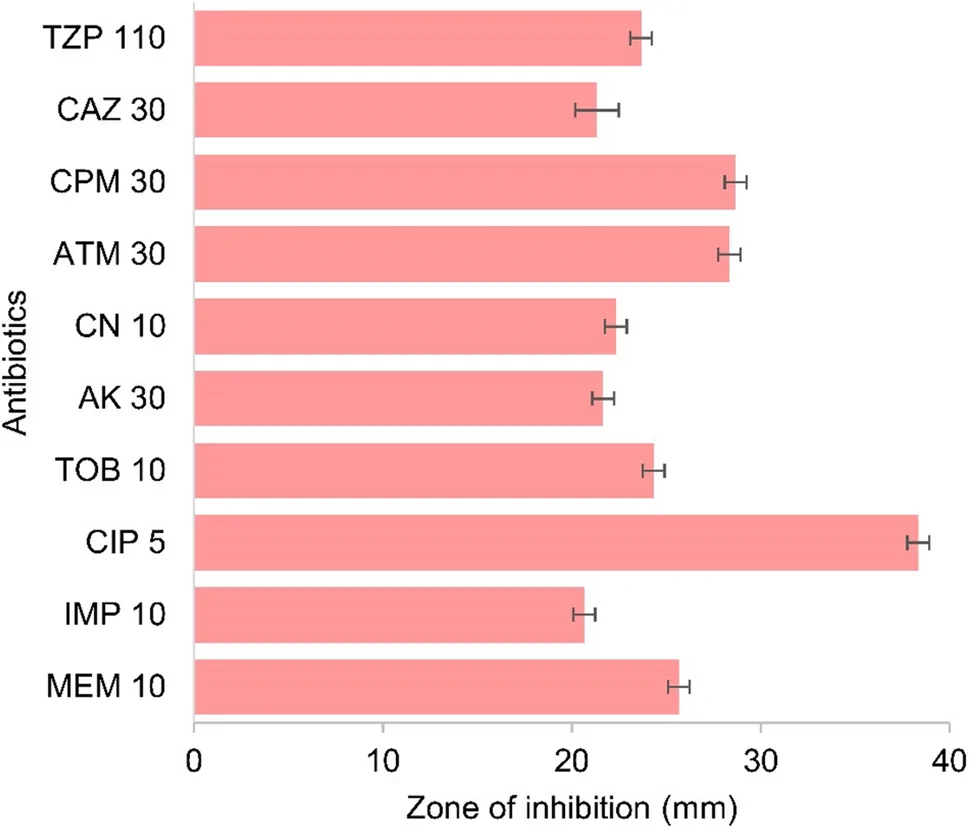
Bar chart illustrating the inhibition zone diameters observed in the antibiotic susceptibility assay of strain CELK12 against ten different antibiotics

The antibiotic susceptibility of *E. adelaidei* CELK12 was evaluated using the disc diffusion method, revealing variable responses to different classes of antibiotics (Fig. 7, Supplementary Fig. S5). Among the tested antibiotics, CELK12 exhibited the highest sensitivity to ciprofloxacin (CIP, 5 µg), with the largest zone of inhibition (38.33 mm). Strong inhibitory effects were also observed for cefepime (CPM, 30 µg; 28.67 mm) and aztreonam (ATM, 30 µg; 28.33 mm), followed by meropenem (MEM, 10 µg; 25.67 mm) and tobramycin (TOB, 10 µg; 24.33 mm). Moderate inhibition was recorded for piperacillin/tazobactam (TZP, 110 µg; 23.67 mm), gentamicin (CN, 10 µg; 22.33 mm), amikacin (AK, 30 µg; 21.67 mm), ceftazidime (CAZ, 30 µg; 21.33 mm), and imipenem (IMP, 10 µg; 20.67 mm). Notably, while genomic analysis identified a limited set of resistance-related determinants, the isolate remained phenotypically sensitive to a wide range of β-lactam, aminoglycoside, fluoroquinolone, and carbapenem antibiotics. This discrepancy indicates that while these genetic elements are part of the isolate’s genetic makeup, they do not confer functional resistance under the experimental conditions. In hemolysis test, CELK12 showed no detectable hemolytic activity on blood agar, with no visible hemolytic zones observed, indicating the absence of α-, or β-hemolysis (Supplementary Fig. S6).

## Discussion

*Enterobacter* species are frequently isolated from a wide range of plant rhizospheres and endophytic niches, where they contribute to nutrient cycling and plant growth promotion [17, 19, 20, 84–86]. The present study offers the first genome-based characterization of an *Enterobacter adelaidei* isolate (strain CELK12) occupying a beneficial, plant-associated ecological niche. The recovery of strain CELK12 from the taro rhizosphere underscores the potential of neglected crop microbiomes as reservoirs for agriculturally relevant microorganisms [87–89]. Furthermore, comparative genomic analysis elucidates the mechanisms by which this specific strain adapts to high-stress, non-clinical environments, revealing a distinct evolutionary trajectory compared to other clinical lineages of the same species [26].

The multi-functional efficacy of CELK12 is not an isolated phenomenon but is fundamentally anchored in its genomic architecture. Our findings reveal a clear convergence between predicted genetic traits and experimental observations. Specifically, critical loci like *gcd*, *pqqC*, and components of the Pho regulon likely constitute a core environmental survival strategy in the taro rhizosphere. In contrast to clinical strains that focus on extracting nutrients from a host organism, the CELK12 genome possesses an enriched set of accessory genes dedicated to mobilizing potassium and phosphate. These traits, further supported through our in vitro biochemical testing, suggest its evolutionary specialization for thriving in nutrient-deficient soil [90, 91]. Additionally, the substantial synthesis of IAA (59.92 µg/mL) is genetically supported by the *ipdC* and *trpE* biosynthetic pathways identified in the PLaBAse analysis [92].

A critical driver of the functional innovation identified in CELK12 is its ability to mitigate abiotic stresses. Marked improvements in the biomass and longitudinal development of rice seedlings treated with CELK12 under saline conditions substantiate the biological significance of genomic attributes linked to oxidative and osmotic resilience (e.g., *katG*, *rpoS*) [93, 94]. Moreover, the ability of CELK12 to preserve seedling vigor under salt stress (up to 150 mM NaCl) corresponds with the genomic integration of the *otsA* (trehalose biosynthesis) and *betA* (glycine betaine) pathways. We posit that the acquisition or retention of these stress-responsive accessory genes represents a refined evolutionary strategy for adapting to the variable salinity levels inherent in taro cultivation systems [95–98]. Nonetheless, subsequent transcriptomic or proteomic investigations are essential to fully elucidate the underlying biochemical mechanisms of the temporal regulation of these characteristics within complex, indigenous soil matrices [99, 100].

Genetic determinants associated with biofilm formation and root colonization, including *csgD* and *fimA*, together with motility-associated genes such as *fliC* and *cheA*, suggest potential ability for surface attachment and directional movement toward root exudates [95, 101–103]. The metabolic versatility of CELK12 is further supported by a repertoire of genes dedicated to the catabolism of plant-sourced carbohydrates, suggesting high ecological adaptability within the rhizosphere environment [104, 105]. PLaBAse predicted plant-microbe interaction factors, such as toxin-related determinants, exopolysaccharide biosynthesis, and adhesion-associated functions, likely enhance microbial competitiveness, biofilm formation, and host association [106]. Conversely, the limited occurrence of plant cell wall-degrading enzymes indicates ecological fitness without strong pathogenic potential [107].

The observed inhibitory effect of CELK12 against *Fusarium concentricum* (47.83%) during in vitro assays is likely supported by diverse biosynthetic and competitive strategies uncovered via genome mining. Computational profiling using antiSMASH pinpointed biosynthetic gene clusters encoding arylpolyenes, siderophores, and RiPPs. Aryl polyenes are known to protect bacteria from oxidative damage that can occur during bacterial-fungal interactions, thereby enhancing survival in competitive ecological niches [108]. Siderophore-producing clusters may exert indirect antifungal pressure by sequestering environmental iron, limiting its availability to the pathogen [109]. Furthermore, the presence of RiPP-related clusters hints at the secretion of bioactive peptides that may contribute to direct antimicrobial activity, though empirical substantiation remains necessary [110]. While genomic projections derived from platforms like antiSMASH and PLaBAse provide significant insights into functional potential, they are inherently speculative and prone to false positives; as sequence-based annotations do not always equate to active gene expression or functional confirmation [111, 112].

Comparative genomic analysis of *E. adelaidei* CELK12 and the reference strain ECC3473 reveals significant ecological divergence despite their taxonomic proximity and conserved core functional profiles (COG, KEGG, and CAZy). While shared metabolic frameworks reflect their close relationship, the limitations of broad functional classifications in resolving niche specialization particularly within genera spanning environmental and clinical lineages necessitate a more granular approach [113–115]. The observed open pan-genome structure (α = 0.11) indicates substantial genome plasticity and suggests that ongoing acquisition of accessory genes may facilitate adaptation to heterogeneous and competitive conditions typical of the rhizosphere. Nevertheless, this pattern warrants a cautious interpretation given the limited genomic sampling currently available. Notably, the unique singleton genes within CELK12 are enriched in determinants for motility, chemotaxis, fimbrial assembly, iron acquisition, and stress tolerance including *cheA/W/Y*, *flg/fli* operons, *fimD/G*, *lpfA/B*,* entB/D*, and oxidative stress-associated genes, essential for rhizosphere persistence and competitive colonization [103, 116]. Genes associated with siderophore-mediated iron uptake, environmental sensing, biofilm-mediated adhesion, and diverse nutrient utilization further reinforces the strain’s ability to thrive in the nutrient-fluctuating matrices of plant-associated habitats. These findings reinforce the consensus that genome plasticity and accessory gene composition, rather than core metabolism, drive the adaptation of closely related *Enterobacter* strains to distinct ecological niches [117, 118].

Considering the clinical significance of several *Enterobacter* species, a rigorous biosafety assessment is essential for evaluating CELK12 as a potential PGPR [119–121]. In this study, we performed a preliminary biosafety assessment based on genomic analysis and selected phenotypic assays. The results suggest that while strain CELK12 retains the genomic features characteristic of environmental *Enterobacter* lineages [85, 86], it possesses a significantly attenuated resistome compared to clinical counterparts such as ECC3473. However, the identification of genes such as *oqxA* and *blaACT-6* in CELK12 genome warrants caution. The observed phenotypic sensitivity, despite the presence of these genetic markers, indicates that they remain ‘cryptic’ or poorly expressed, potentially due to the absence of specific induction signals or functional promoters [122, 123]. Crucially, genomic mining revealed that these resistance factors are located on the chromosome instead of on plasmids or within known transposons and integrons, which may restrict their rapid dissemination via horizontal gene transfer (HGT) to neighboring rhizospheric microbial communities. Furthermore, the absence of global regulatory elements such as *marA* and *ramA* suggests that CELK12 is devoid of high-level machinery typical of multidrug-resistant clinical lineages [124, 125]. However, considering the characteristic fluidity of *Enterobacter* genomes, the presence of these genes implies a latent potential for resistance that requires vigilant observation. Furthermore, extensive multi-site field evaluations and more comprehensive sampling are essential to fully assess the strain’s long-term stability and its broader potential as a bio-based agricultural input.

## Conclusion

This study provides the first genome-resolved evidence of *Enterobacter adelaidei* (strain CELK12) occupying a beneficial, plant-associated niche, expanding the known ecological breadth of a species previously associated primarily with clinical settings. By demonstrating that the isolate *E. adelaidei* CELK12 possesses a specialized genomic suite for plant-beneficial interactions under the conditions examined, these findings provide a more refined, ecologically centered perspective for evaluating the functional diversity of *Enterobacter* variants in the context of agricultural biotechnology. Our data suggest that niche specialization within this species is likely a strain-dependent occurrence facilitated by accessory genome fluidity, which must be evaluated at the isolate level through the lens of genomic innovation.

## Supplementary Information

Supplementary Material 1.

Supplementary Material 2.

## Data Availability

The whole genome sequence of *Enterobacter adelaidei* CELK12 has been submitted to NCBI under Bioproject ID: PRJNA1344764 and Biosample ID: SAMN54939169. The genome assembly of the bacterial isolate is publicly available in NCBI GenBank database under the accession number JBUBKU000000000.
