# Supplementary material for: Comparative genomic insights into a plant-associated Enterobacter adelaidei strain isolated from the taro rhizosphere
Source: BMC Genomics. 2026 Jun 17;27:709. doi: 10.1186/s12864-026-13081-3 (PMC13508372; doi:10.1186/s12864-026-13081-3)
Supplement: Supplementary file 1 [file 12864_2026_13081_MOESM1_ESM.pdf]

Supplementary Figures

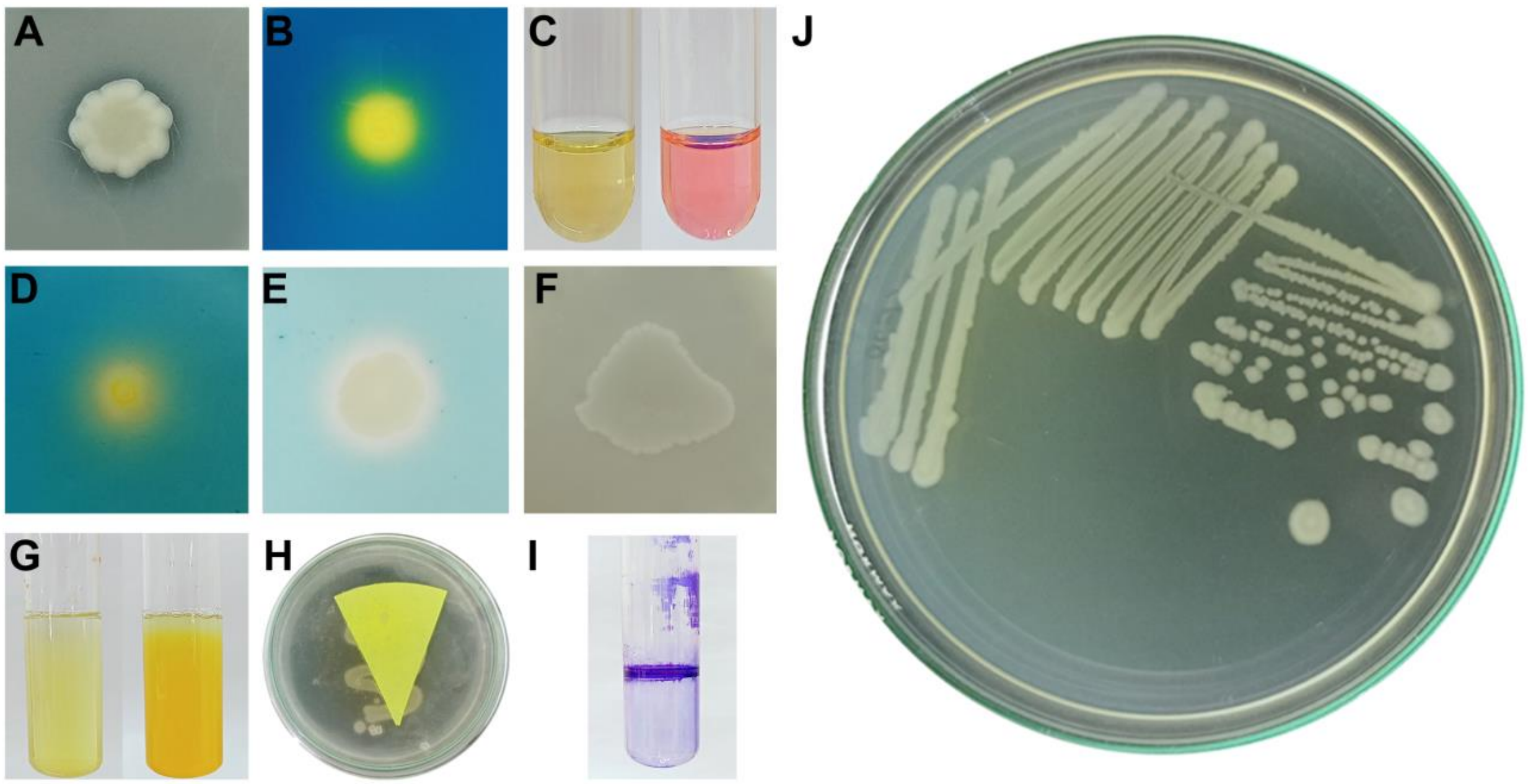

Supplementary Fig. S1. *In vitro* plant growth-promoting (PGP) activities of strain CELK12. (**A)** P solubilization. (**B)** K solubilization. (**C)** IAA biosynthesis. (**D)** Nitrogen fixation. **(E)** Siderophore production. **(F)** Protease production. (**G)** Ammonia formation. (**H)** HCN production. (**I)** Biofilm formation. (**J**) Growth of CELK12 on nutrient agar plate.

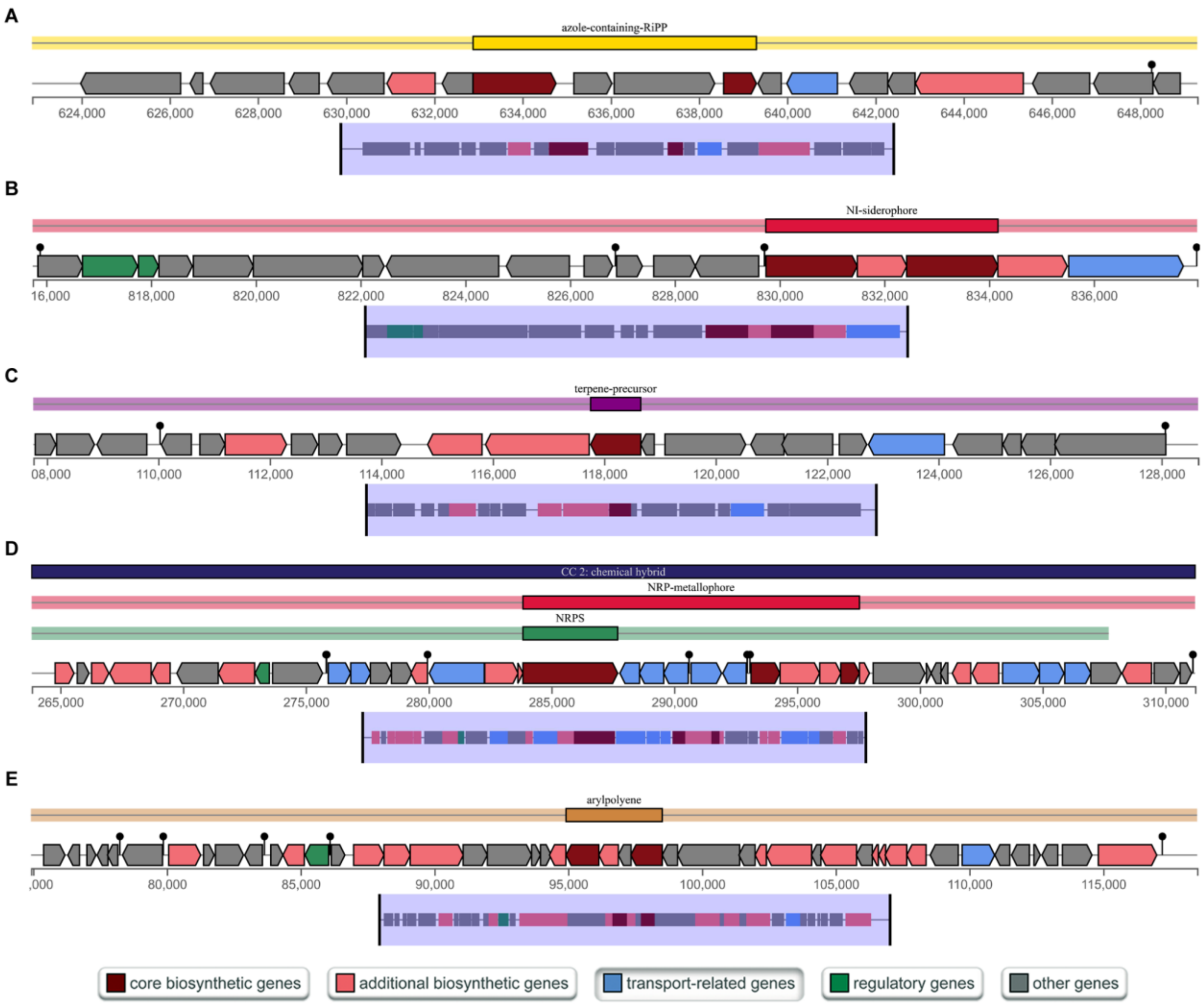

Supplementary Fig. S2. AntiSMASH predicted biosynthetic gene clusters of strain *E. adelaidei* CELK12. (**A**) Azole-containing-RiPP. (**B**) NI-siderophore. (**C**) Terpene-precursor. (**D**) NRP-metallophore, NRPS. (**E**) Arylpolyene.

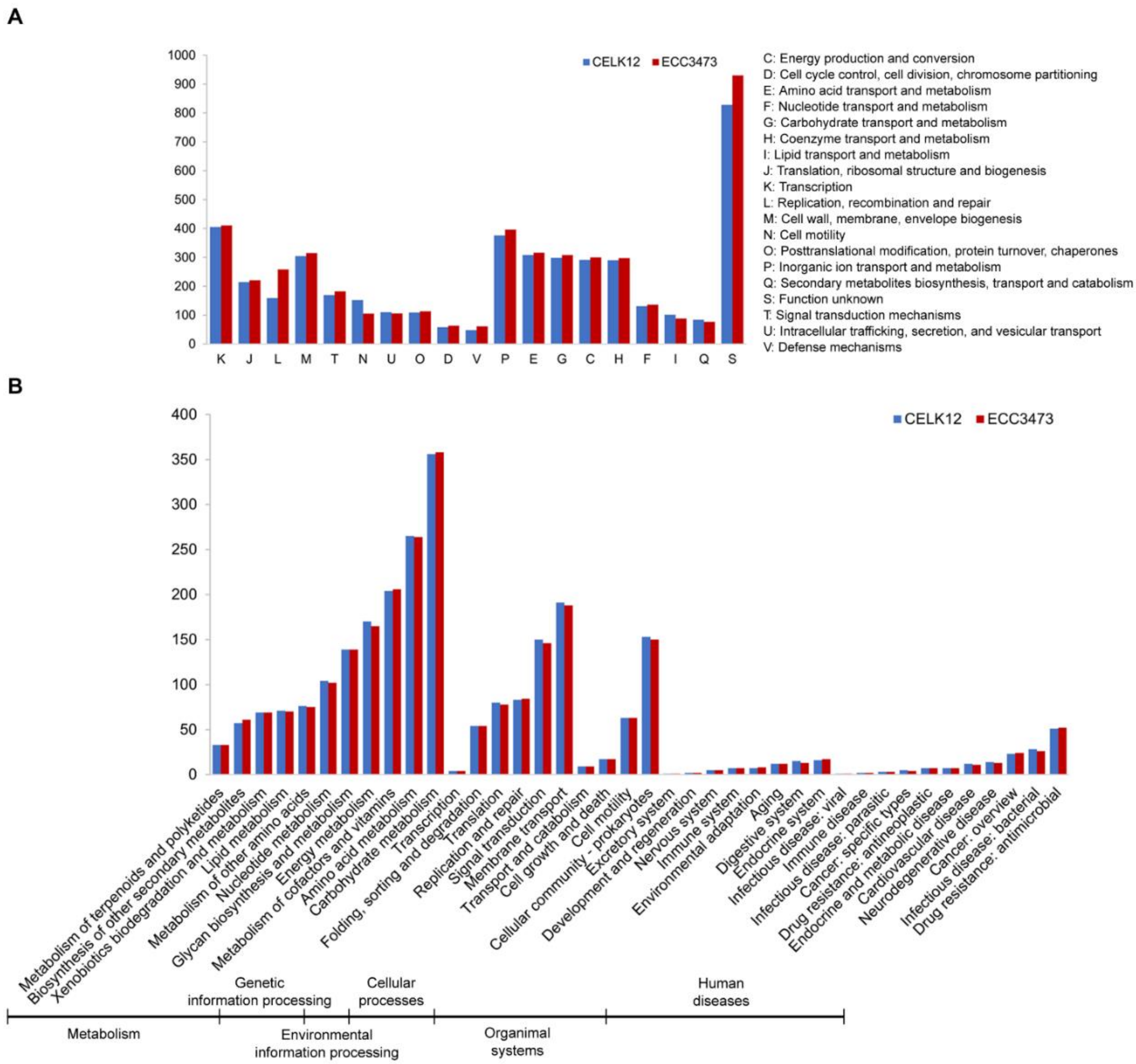

Supplementary Fig. S3. Comparative functional category distribution of *E. adelaidei* CELK12 and the reference strain *E. adelaidei* ECC3473 based on genome annotation. (**A**) COG functional classification. (**B**) KEGG pathway classification.

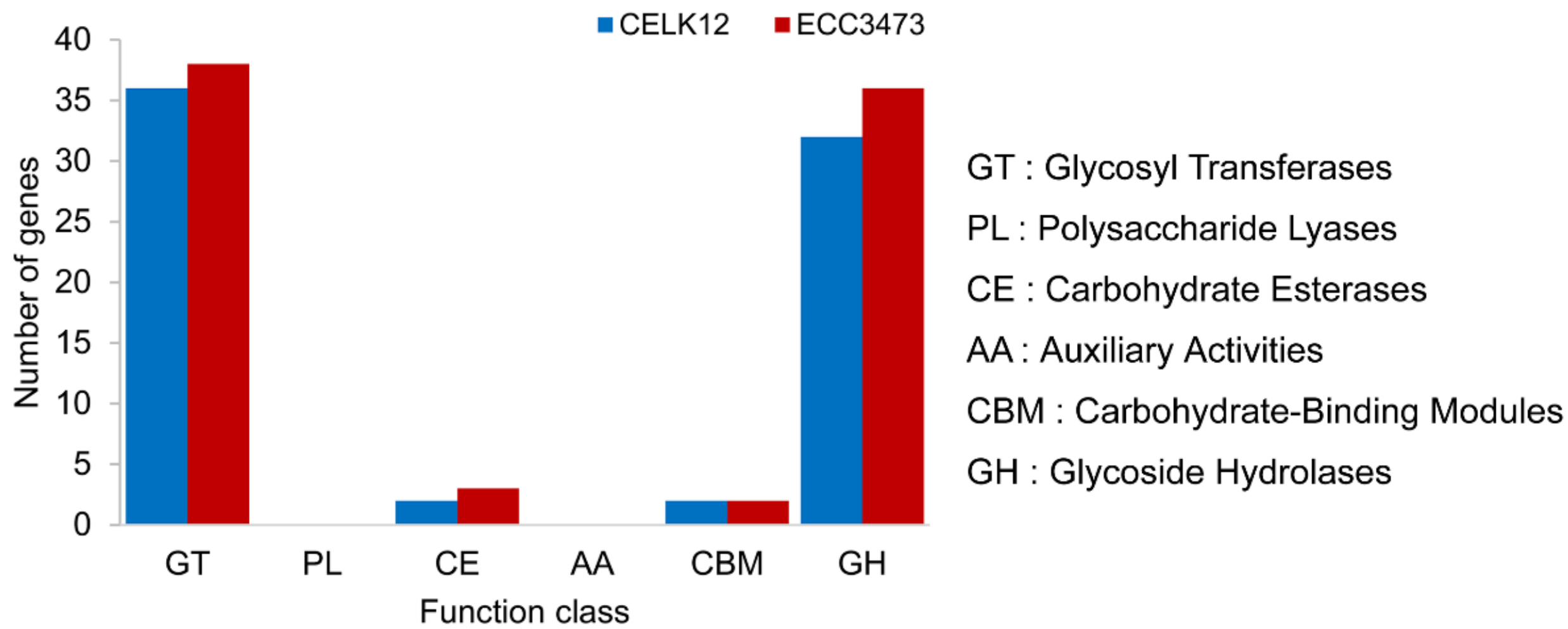

Supplementary Fig. S4. Bar charts illustrating the distribution of carbohydrate-active enzymes (CAZymes) within the *E. adelaidei* CELK12 and *E. adelaidei* ECC3473 genome.

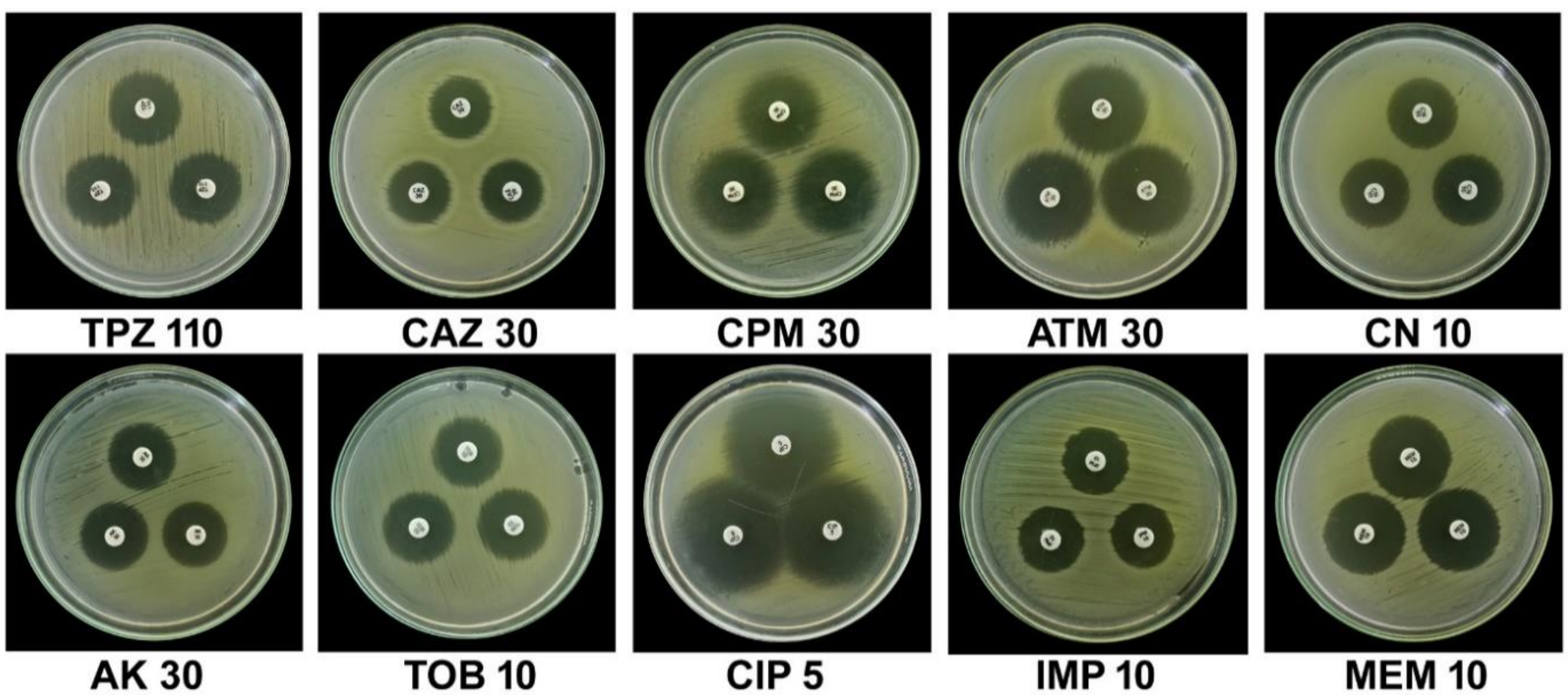

Supplementary Fig. S5. Muller-Hinton agar plates demonstrating inhibition zones produced by ten different antibiotics against *E. adelaidei* CELK12 in disc diffusion assay.

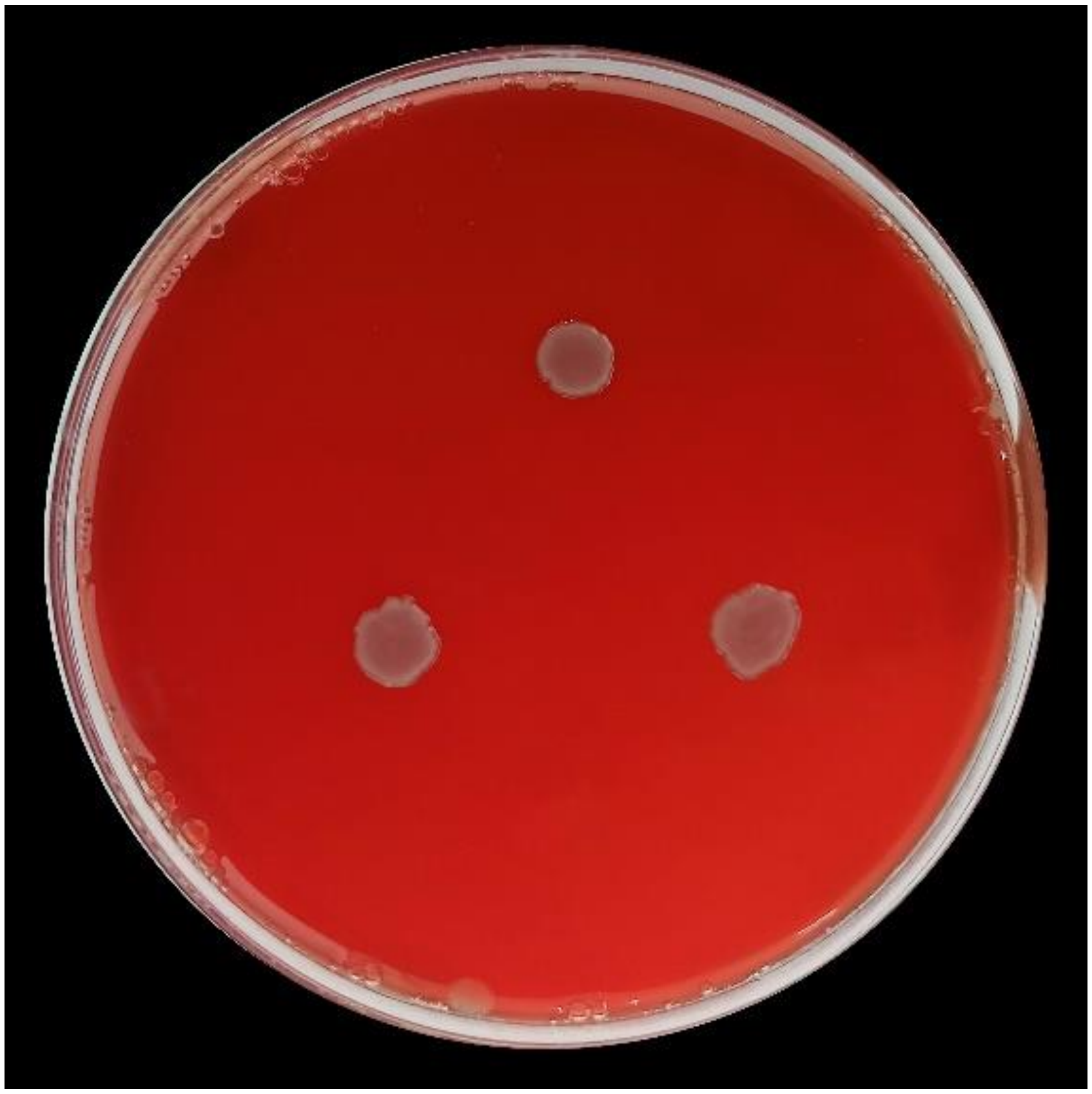

Supplementary Fig. S6. Blood agar plate indicating the absence of a hemolytic zone in the hemolysis assay of isolate *E. adelaidei* CELK12.
